# Computational Model of Membrane Fission Catalyzed by ESCRT-III

**DOI:** 10.1371/journal.pcbi.1000575

**Published:** 2009-11-20

**Authors:** Gur Fabrikant, Suman Lata, James D. Riches, John A. G. Briggs, Winfried Weissenhorn, Michael M. Kozlov

**Affiliations:** 1 Department of Physiology and Pharmacology, Sackler Faculty of Medicine, Tel Aviv University, Tel Aviv, Israel; 2 Unit of Virus Host Cell Interactions (UVHCI) UMR 5233 Université Joseph Fourier-EMBL-CNRS, Grenoble, France; 3 Structural and Computational Biology Unit, European Molecular Biology Laboratory, Heidelberg, Germany; Stanford University, United States of America

## Abstract

ESCRT-III proteins catalyze membrane fission during multi vesicular body biogenesis, budding of some enveloped viruses and cell division. We suggest and analyze a novel mechanism of membrane fission by the mammalian ESCRT-III subunits CHMP2 and CHMP3. We propose that the CHMP2-CHMP3 complexes self-assemble into hemi-spherical dome-like structures within the necks of the initial membrane buds generated by CHMP4 filaments. The dome formation is accompanied by the membrane attachment to the dome surface, which drives narrowing of the membrane neck and accumulation of the elastic stresses leading, ultimately, to the neck fission. Based on the bending elastic model of lipid bilayers, we determine the degree of the membrane attachment to the dome enabling the neck fission and compute the required values of the protein-membrane binding energy. We estimate the feasible values of this energy and predict a high efficiency for the CHMP2-CHMP3 complexes in mediating membrane fission. We support the computational model by electron tomography imaging of CHMP2-CHMP3 assemblies *in vitro*. We predict a high efficiency for the CHMP2-CHMP3 complexes in mediating membrane fission.

## Author Summary

Membrane fission is a key step of fundamental intracellular processes such as endocytosis, membrane trafficking, cytokinesis and virus budding. The fission reaction requires substantial energy inputs provided by specialized proteins. Recently, the ESCRT-III proteins have been implicated in membrane budding and fission involved in multivesicular body formation, cytokinesis and virus budding. The ESCRT-III proteins self-assemble into circular filaments and flat spirals in the membrane plane and generate tubular structures with dome-like end caps. We suggest and elaborate computationally on a mechanism by which the ESCRT-III complexes can drive membrane fission. The essence of the mechanism is in generation in the course of membrane attachment to the dome-like surface of an ESCRT-III assembly of a thin membrane neck accumulating large elastic stresses. Relaxation of these stresses can drive the neck fission and formation of separate vesicles of biologically relevant sizes. Estimations of the membrane affinity to the protein surface required for the neck fission to occur and comparison of these values with the experimentally expected values justify quantitatively the proposed mechanism and demonstrate that ESCRT-III assemblies must be highly effective in promoting membrane fission.

## Introduction

### Membrane shaping and fission by proteins

Membrane fission leading to division of one continuous membrane into two separate ones is ubiquitous in cell physiology. It is one of the crucial events in generation of transport intermediates from plasma membranes and intracellular organelles; steady-state dynamics of the endoplasmic reticulum, mitochondria and Golgi complex; virus budding, cytokinesis and other fundamental phenomena (see for review e.g. [1]–[3]).

In the process of fission, a membrane changes its shape and undergoes a topological transformation which includes transient perturbations of the membrane continuity. To overcome the membrane resistance to shaping and remodeling, a substantial energy has to be invested into the system, which requires action of specialized proteins (see for review [2],[3]). Identification of proteins which shape and remodel membranes in the course of diverse intracellular processes has become a hot topic of cell biology [1],[3],[4]. The major advance has been achieved in discovering proteins generating and/or sensing the membrane curvature. The list of such proteins is constantly expanding and the mechanisms of their action are being elaborated [1],[4],[5]. Less progress has been made in understanding how proteins drive the membrane fission *per se*. While several protein types such as the dynamin-family proteins (see e.g. [6]–[10]), CtBP1/BARS [11] and PKD [12] have been implicated in fission of cell membranes, until recently, the ability to split membranes was unambiguously demonstrated for, perhaps, only one protein, dynamin-1 [9], [13]–[15]. Whereas different versions of the mechanism of membrane fission by dynamin-1 were suggested (see for review [10]), the idea unifying the majority of these proposals is that dynamin self-assembles on the membrane surface into helical oligomers constricting the membrane underneath into thin tubes. Strong mechanical stresses induced by dynamin in the tubulated membrane upon GTP hydrolysis can relax as a result of membrane division and, therefore, drive membrane fission.

### Membrane remodeling by ESCRT-III complexes

Accumulating evidence suggests that the ESCRT (Endosmal Sorting Complexes Required for Transport) complexes [16] – are able to catalyze the membrane budding and fission processes. The ESCRT machinery consists of five different complexes - theVps27complex (ESCRT-0), ESCRT-I, -II, and -III, and the Vps4 complex - whose coordinated action sorts trans-membrane proteins into intralumenal vesicles (ILV), which bud off from the limiting membranes of endosomes and transform endosomes into multivesicular bodies (MVB) [16]–[19].

In addition to the MVB generation, the combined action of ESCRT-III and VPS4 complexes are required for the budding of some enveloped viruses including HIV-1 [20]and during late steps in cytokinesis [21]–[24]. It is thus most likely that ESCRT-III and VPS4 catalyze membrane fission reactions, common to all three biological processes [21]–[25].

The ESCRT-III complex in yeast consists of four core subunits Vps20, Snf7, Vps24, and Vps2 [26] whose mammalian analogues are the charged multivesicular body proteins CHMP6, CHMP4, CHMP3 and CHMP2, respectively. The subunits are consecutively recruited to the membrane in the order of Vps20/CHMP6, Snf7/CHMP4, Vps24/CHMP3 and Vps2/CHMP2 [27]–[29] and their assembly into higher order complexes was suggested to drive the inward membrane budding in vitro [28]. Moreover, these four proteins are able to act as minimal budding machinery as was confirmed by demonstration that their sequential addition to giant unilamellar vesicles (GUV) generated membrane invagination and abscission of the inward vesicles [29]. Specifically, formation of membrane buds connected by open necks to the initial membrane was shown to depend, critically, on the Snf7(CHMP4) and Vps20(CHMP6) subunits, while the neck fission proved to require the Vps24(CHMP3) subunits [29].

### Mechanistic links between the structures of the ESCRT-III assemblies and the membrane remodeling

Three different albeit similar models for ESCRT-III catalyzed budding have been suggested [30]. First, Snf7 (CHMP4) circular filaments or flat spirals lying in the membrane plane [31] start at the center of a newly formed membrane bud and catalyze membrane bending as the bud grows [31]. A second model suggests that a circular ESCRT-III filament with asymmetric ends delineates a membrane patch containing cargo molecules and constricts the neck of an evolving membrane bud via the disassembly action of Vps4 [27]. A third model, similar to the second one, proposes that an ESCRT-III spiral surrounds and constricts a cargo containing membrane domain leading to membrane budding and fission [29]. However, spiral polymers of ESCRT-III have only been observed for hSnf7(CHMP4) in vivo [31] and in vitro [32], whereas the detachment of the forming vesicle including fission of a membrane neck was shown to be crucially dependent on Vps24(CHMP3) [29]. Therefore, in addition to the Snf7(CHMP4) filaments, the structures formed by self-assembly of Vps24(CHMP3) must play an indispensable role in the ESCRT-III mediated membrane budding and fission.

CHMP3 (Vps24) and CHMP2A (Vps2) form heterodimers [26],[33] that assemble into tubular nano-structures which display a variety of end-cap shapes including nearly hemispherical dome-like end-caps ([34] and the section “Experimental support for the model” below). The external and internal radii of these structures are approximately 52 and 43nm, respectively [34]. In vitro, the AAA ATPase VPS4 binds to the inside of the CHMP2-CHMP3 polymers and leads to their disassembly in the presence of ATP [34]. The external surface of a CHMP2-CHMP3 nano-structure has a considerable affinity to membranes containing acidic lipids [34]. Therefore, in the process of self-assembly, the CHMP2-CHMP3 complex must be able to attract a lipid bilayer, hence, scaffolding the bilayer into a strongly curved shape, a process that might drive membrane fission reactions [34].

### Specific features of the ESCRT-III- mediated membrane fission

In spite of the apparent similarities between the dynamin-I and CHMP2-CHMP3 assemblies such as (i) the ability to scaffold membranes into cylindrical shapes, and (ii) the energy input by nucleotide hydrolysis, CHMPs cannot employ any of the mechanisms of membrane fission suggested for the dynamin action. Indeed, topologically, the fission reactions mediated by dynamin and ESCRT-III are directed differently: dynamin and its partners drive membrane budding and abscission towards the cytosol, while ESCRT-III mediates membrane abscission away from the cytosol and towards the lumen of an endosome. Structurally, a membrane portion tubulated by a dynamin oligomer is situated within the protein scaffold and, hence, could undergo further thinning upon detachment from dynamin and divide by self-fusion within the protein framework [14]. In contrast, the membrane wrapped around a CHMP2-CHMP3 structure is attached to the outside surface of the protein scaffold and, hence, the scaffold hinders the membrane sterically from direct thinning and self-fusion. Thus, the character of membrane deformation leading to fission driven by CHMP2-CHMP3 structure must differ essentially from that generated by dynamin and the mechanics of the fission reaction must be dissimilar in the two cases.

Here, we suggest and integrate the current structural knowledge on ESCRT-III complexes to elaborate on a novel mechanism of membrane fission by dome-like assemblies formed by the CHMP2-CHMP3 subunits of ESCRT-III. The essence of our proposal is that, in contrast to the fission mechanisms suggested for the dynamin action (see for review [10]), the site of membrane fission driven by ESCRT-III is not co-localized with the protein scaffold but rather emerges aside of it within a membrane neck which forms in the course of membrane wrapping around the ESCRT-III dome. The major energy for the fission reaction comes from the energy of membrane attachment to the surface of the ESCRT-III complex. We discuss a possibility for a reinforcement of the ESCRT-III based mechanism by the Vps4 binding.

Our calculations predict that ESCRT-III domes can serve as effective mediators of membrane fission resulting in generation of vesicles of biologically relevant dimensions.

## Model

**Figure 1:**
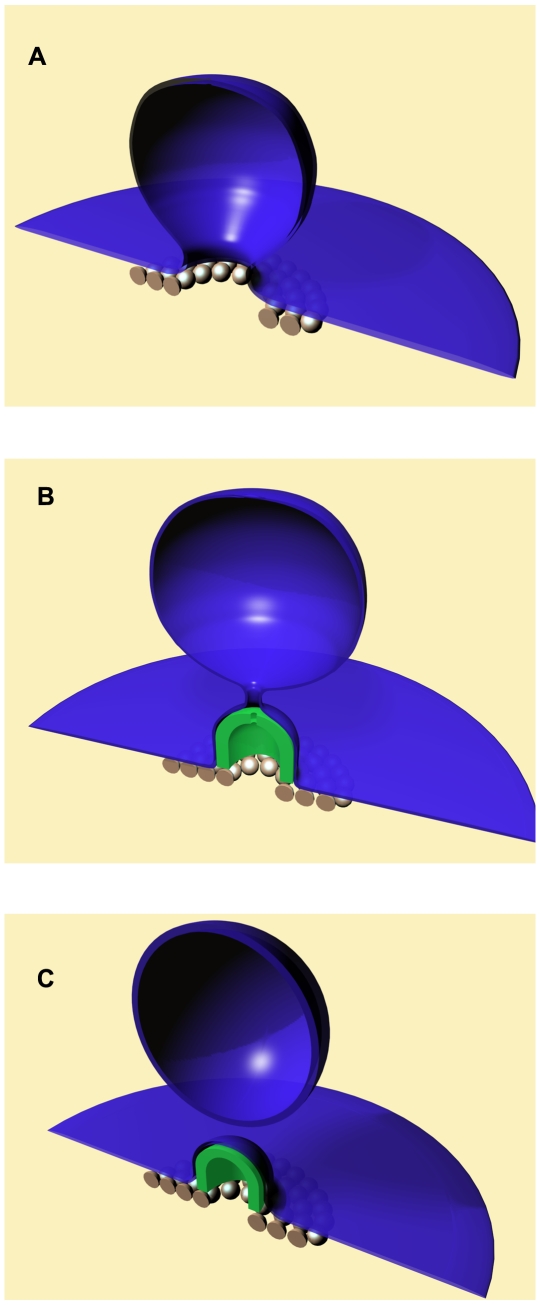
Model for membrane budding and fission by the ESCRT-III sub-complexes. A. Formation of the initial bud by CHMP4(Snf7) as suggested in [27],[29]. B. Self-assembly of CHMP2-CHMP3 nanotube with a dome-like end-cap. C. Fission of the neck and completion of the vesicle formation.

We propose the following scenario for the membrane budding and fission by ESCRT-III complexes. At the first stage, the CHMP4 subunits are recruited to the membrane via CHMP6 [28] and self-assemble on the membrane surface into a circular filament or flat spiral [31], which leads to sequestering of a membrane patch and its bending into an initial bud, as proposed in [29] and illustrated in (Fig. 1a). We assume that the area of the initial bud sequestered by the CHMP4 spiral remains constant in the course of all downstream processes. In fact, attachment of the CHMP4 oligomers to the membrane surface [31] evidences a considerable attractive interaction between the CHMP4 and the lipid polar head groups. The lipid molecules whose head groups are bound to the protein spiral along the periphery of the bud (Fig. 1a) must build an effective “fence” preventing, within the time scale of membrane fission, the lipid exchange between the bud and the surrounding membrane, and, hence, restricting the changes of the bud area.

Next, the CHMP2A and CHMP3 subunits start self-assembling within the neck of this initial membrane bud which is accompanied by a concomitant attachment of the membrane to the emerging protein complex (Fig. 1b). The attachment is mediated by the attractive membrane-protein interaction. The total area of the initial bud is assumed to exceed considerably the area of the CHMP2-CHMP3 complex even after completion of its assembly. As a result, only a portion of the initial bud membrane can be directly attached to the protein structure (Fig. 1b). The rest of the membrane remains free and is connected by a neck to the attached membrane (Fig. 1b).

In the course of self-assembly, the CHMP2A-CHMP3 polymer builds up a tube whose end-cap gradually closes into a nearly hemi-spherical dome-like shape (Fig. 1b). The larger the fraction of the protein dome is assembled and covered by the membrane the thinner the neck. The neck tightening is accompanied by an increasing bending of its membrane and the related accumulation of the membrane elastic energy [35].

At a certain stage, the membrane elastic energy accumulated within the neck becomes so large that its relaxation can drive the neck scission, which results in formation of a spherical vesicle and a membrane cap covering the CHMP2A-CHMP3 dome (Fig. 1c). Two requirements have to be satisfied for fission to occur. First, the membrane scission event has to be overall energetically favorable meaning that the total energy of the system before fission must exceed the energy of the post-fission vesicle and membrane cap attached to the ESCRT-III dome. Fulfillment of this condition ensures the general feasibility of the fission reaction but does not guarantee that the reaction will be sufficiently fast to make it biologically relevant. The second requirement concerns the fission rate which can be limited by the energy barriers. According to this requirement, the energy barriers produced by the intermediate structures formed in the course of membrane splitting have to vanish or remain small. Based on electroporation experiments, feasible energy barriers which can be overcome within a time scale of few seconds by a membrane of large area is about 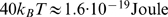, (where 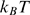 is the product of the Boltzmann constant and the absolute temperature) [2]). For small membrane fragment making up a membrane neck, the feasible energy barrier must be a few times lower and constitute less than 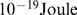. A major energy barrier is related to the strongly deformed intermediate structures forming transiently in the course of the process. In analogy to the well understood process of membrane fusion (see for review [36]–[38]), we assume that this energy barrier is associated with the hemi-fission intermediate in which the internal monolayer of the membrane neck is already split, while the second monolayer is still intact [35]. According to the analysis of fission of a membrane neck emerging during membrane budding by a spherical coat, the fission reaction is energetically favorable and the hemi-fission intermediate does not represent a kinetic barrier if the membrane neck in its thinnest cross-section narrows down to the threshold radius of about 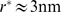 [35].

The attractive interaction between the subunits of the CHMP2-CHMP3 structure must be much stronger than all other relevant interactions characterizing the system. According to the results below for a characteristic energy needed to bend the membrane around the protein dome (∼0.25 mN/m) and a characteristic area of about 22.5 nm^2^ exposed by one CHMP protomer to interaction with the membrane [33], the low limit for the energy of the subunit interaction needed for the protein structure to remain stable upon bending of the attaching membrane, can be estimated as 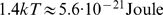. In reality, the interaction energy of the CHMP protomer must exceed considerably this estimate since their self assembly is, practically, irreversible [34]. Based on this assumption, we propose that the protein self-assembly proceeds irrespectively of the membrane attachment, while the latter follows the dome formation and its extent is determined by the interplay between the membrane bending energy and the membrane affinity to the protein surface.

In the following, we will analyze quantitatively the above scenario of the membrane neck fission by the CHMP2-CHMP3 dome. Since the thinning of the membrane neck is driven by the progressing membrane attachment to the protein dome, we will consider only the dome part of the protein complex. We will compute the extent of the ESCRT-III dome coverage by the membrane and the corresponding shapes of the membrane bud for different values of the membrane affinity to the ESCRT-III complex. We will find the affinity values at which the membrane neck becomes sufficiently narrow to favor energetically the fission reaction. We will also determine the affinity required to reach the threshold neck radii at which the energy barrier associated with the hemi-fission intermediate becomes negligible and does not limit the fission rate.

### Main definitions and equations

**Figure 2:**
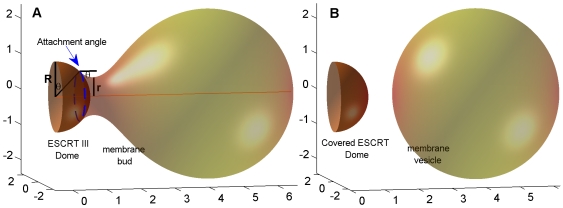
Lipid membrane attached to a protein dome – computed configuration and definitions. (A) Fore-fission state: 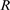
- radius of the protein dome surface 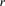 - the neck radius, 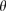
- the attachment angle. (B) Post-fission state. The total membrane area 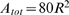.

We consider a hemi-spherical protein dome of radius 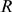 serving as a scaffold for attachment of a membrane fragment of a total area 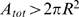 (Fig. 2a). While, in reality, the membrane attachment to the dome proceeds concomitantly with the dome assembly, for the calculation purposes we will regard the dome to be completed. This is based on a plausible assumption that the attractive interaction between the subunits of the CHMP2-CHMP3 structure must be much stronger than all other relevant interactions characterizing the system. Therefore, the protein self-assembly proceeds irrespectively of the membrane attachment, while the latter follows the dome building and its extent is determined by the interplay between the membrane bending energy and the membrane affinity to the protein surface.

The absolute value of the energy of the membrane interaction with the dome surface per unit area of the membrane-protein interface will be referred to as the membrane affinity and denoted by 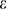. Since the membrane-protein interaction is attractive its energy is negative and its value per unit area is 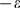. Note that, according to our definition, the affinity 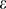 accounts only for the direct (probably, electrostatic) interaction between the protein and the lipid polar groups and does not include the energy of membrane bending, which accompanies the membrane binding to the protein dome and contributes to the total energy of this process. Therefore, the value of 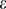 is not supposed to depend on curvature of the protein surface. In this respect, the notion of the affinity we are using differs from the total energy of the membrane attachment to the protein complex, which includes the bending contribution and is commonly used to characterize interaction of proteins with bent membranes (see e.g. [1],[4],[39],[40]). In our approach the curvature effects are considered separately from the direct membrane-protein interaction.

The membrane adopts a curved shape of a bud characterized at each point by the total curvature 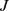 and the Gaussian curvature 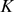 [41]. The radius of the narrowest cross-section of the bud neck will be referred to as the neck radius, 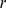 (Fig. 2a). The membrane bending energy per unit area of the membrane mid plane, 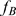, is given by [42],[43],
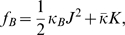
(1)where 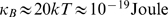 is the bilayer bending modulus (see e.g. [44]), and 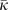 is the bilayer modulus of Gaussian curvature whose values were not directly measured but estimated to be negative (see e.g. [45],[46]).

We analyze two alternative states of the system: the fore-fission state where the membrane bud is connected by a membrane neck to the membrane portion attached to the protein dome (Fig. 2a), and the post-fission state represented by a separate spherical vesicle and the protein dome completely covered by the membrane (Fig. 2b). Our goals are (i) to compute the energies of the two states and to find, by their comparison, the affinity values 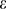 at which the membrane fission event is energetically favorable, and (ii) to determine 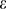 at which the membrane neck in the fore-fission state becomes as small as 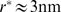 guaranteeing fast fission [35].

In the fore-fission state, the extent of the membrane attachment to the protein dome will be characterized by the angle 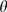 referred below to as the attachment angle which indicates the position of the upper border of the attached area 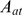 (Fig. 2a). The total energy of the system in the fore-fission state, 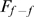, is the sum of two contributions. First, the total attachment energy 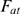 found by integration of the attachment energy density, 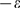, over the attached area 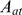. Second, the total bending energy of the membrane, 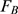, determined by integration of 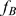 over the whole area of the membrane including 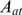 and the area of the bud 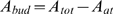. Taking into account Eq.1 and the system geometry (Fig. 2a), the total energy of the fore-fission state can be expressed as
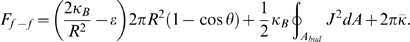
(2)


The first contribution to the Eq. 2 represents the sum of the attachment energy 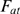 and the bending energy of the attached membrane portion whose total curvature, 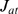, is related to the dome radius, 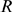, by 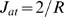. The second contribution is the bending energy of the bud, which depends on the curvature distribution along the bud surface. The third contribution is the energy of the Gaussian curvature, which does not depend on the system configuration. The energy (Eq. 2) has to be minimized with respect to the attachment angle 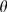 and the distribution of the total curvature 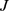 along the surface of the bud for any given value of the affinity 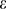. This will give the equilibrium values for 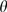 and the corresponding attached area 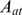, determine the equilibrium shape of the membrane bud including its neck radius 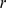, and provide the equilibrium total energy of the fore-fission state. Because of a complex shape of the membrane bud, minimization of Eq.2 will be performed numerically by the standard method of finite elements using the COMSOL Multiphysics software.

In the post-fission state, consisting of a spherical vesicle and the hemi-spherical dome covered completely by the membrane (Fig. 2b) the total energy is
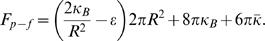
(3)


In the following, we can skip the Gaussian curvature contribution to the fore-fission energy 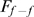, and account for the addition of 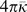 to the energy of the post-fission state 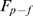.

### Materials and methods

CHMP2A/CHMP3 polymers were assembled and analyzed by negative staining electron microscopy as described [34]. CHMP2A/CHMP3 polymers were applied to a holey carbon grid and plunge frozen in liquid ethane. The samples were examined in an FEI F30 Polara microscope, equipped with a Gatan GIF post-column energy filter [47]. Tilt series were acquired over an angular range of 120 degrees, at a nominal magnification of 27,500 times, which corresponded to a pixel size of 0.49nm, and at a defocus of 5 to 7 microns. Tomograms were generated from these tilt series using the IMOD software package [48] and visualized in Amira (Visage Imaging).

## Results

We consider the membrane affinity, 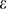, as the major parameter determining the system configurations and the conditions for membrane fission. Other parameters whose values may vary for different membranes are the membrane area 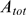 and the membrane modulus of the Gaussian curvature, 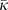. For 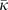 we consider the range 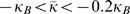 [45],[46]. The range of the membrane area is chosen to be 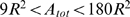, where 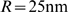 is the external radius of the dome surface. This corresponds to variation of the vesicle diameters in the post-fission state in the biologically relevant range between 20 nm and 100 nm.

### Fore-fission state

**Figure 3:**
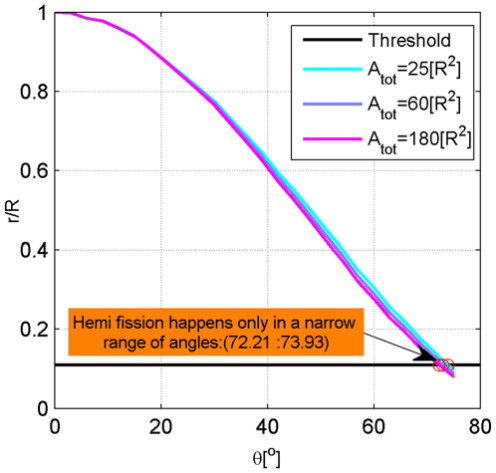
Dependence of the neck radius on the attachment angle. The lines correspond to different values of the membrane area. (1) 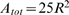; (2) 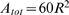; (3) 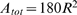.

A typical computed shape of the membrane bud corresponding to a certain attachment angle 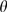, is presented in Fig. 2a and can be described as a sphere-like cap connected to the attached membrane by a funnel-like neck. The larger the angle 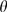, the smaller the neck radius 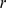 (Fig. 3). At the attachment angle 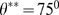 the neck radius becomes smaller than the threshold value, 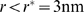, which fulfills the condition of the fast fission [35]. Therefore, we limited the considered range of the attachment angles by 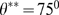. Generally, the computation could be stretched to higher attachment angles corresponding to even narrower necks. This would require, however, including in the elastic energy model additional terms of higher order in the curvature of the internal monolayer of the neck, and taking into account the energy of the short range hydration repulsion through the neck lumen between the elements of the internal surface of the neck. Such sophistication of the model would complicate considerably the computation without significant changes of the model predictions on the neck fission.

**Figure 4:**
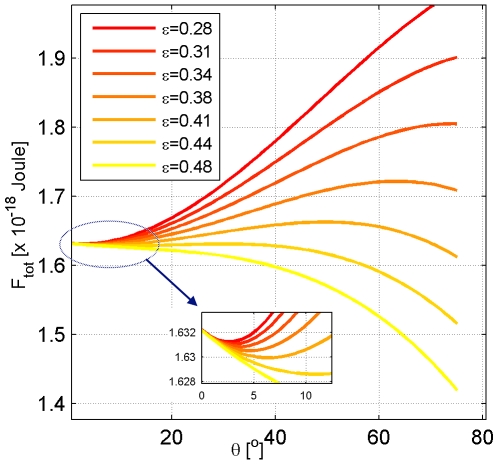
Dependence of the total system energy on the attachment angle. The lines correspond to different values of the membrane affinity to the protein dome surface 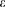 whose values are presented in the insert in mN/m, the total membrane area is 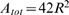.

The character of the dependence of the system energy 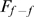 on the attachment angle 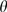 is determined by the affinity 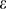 (Fig. 4). According to the first term in Eq.2, the membrane binding to the protein dome will occur only if the affinity exceeds a certain value, 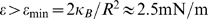, which is the least affinity needed for compensation of the energy penalty of membrane bending accompanying the attachment to the dome surface.

**Figure 5:**
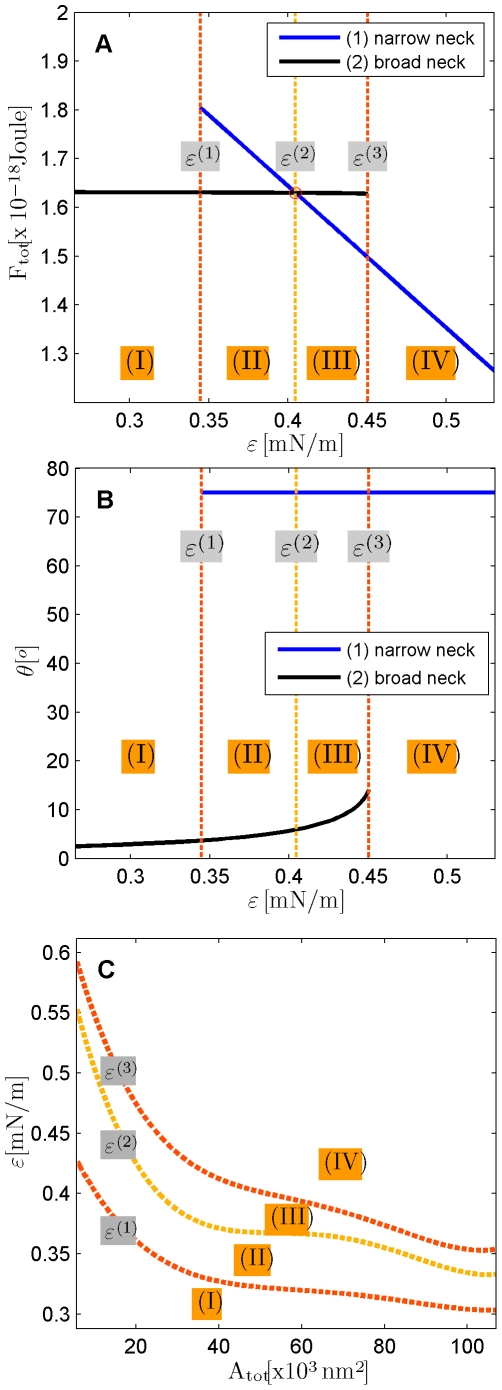
Phase diagrams describing different regimes of the system configurations in the fore-fission state. The phase boundaries are determined by the characteristic values of the membrane affinity to the protein dome surface, 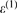, 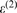, and 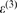. (A) The total energies of the narrow- and broad- neck configurations are represented by the lines 1 and 2, respectively. (B) The attachment angles in the narrow and broad neck configurations are represented by the lines 1 and 2, respectively, 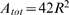 (C) The characteristic affinities depending on the total membrane area 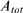. The phase diagrams are divided into four regions corresponding to different regimes of the possible configurations of the system: (I) only broad neck; (II) stable broad neck and quasi-stable narrow neck; (III) stable narrow neck and quasi-stable broad neck; (IV) only narrow neck.

At each particular affinity value 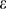 larger than 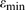, the system can reside in a stable or quasi-stable configuration described by the values of 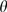 corresponding to the energy minima (Fig. 4). There are four different ranges of the affinity 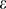 determining different regimes of the possible system configurations. Transitions between these regimes are determined by the three characteristic values of the affinity denoted by 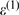, 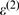 and 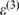 and presented in Fig. 5.

The first regime corresponds to the affinities smaller than the first characteristic value, 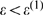. Here, the energy has one minimum at small values, 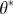, of the attachment angle (Fig. 4), meaning that the stable configuration of the system is a bud with a neck whose radius 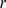 is somewhat smaller than but comparable with the radius of the protein dome 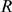. We will refer to this configuration as the broad neck configuration.

In the second regime, the affinity varies between the first and the second characteristic values, 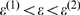. In this range, a second energy minimum emerges at the largest possible attachment angle within the considered range, 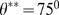 (Fig. 4), corresponding to a bud with a neck of radius 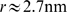 (Fig. 3). This configuration will be called the narrow neck configuration. The total energy in the second minimum is higher than in the first one, 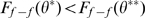, which means that the narrow neck is a quasi-stable while the broad neck is a stable configuration. It has to be noted that, in contrast to the first energy minimum, the second one is not characterized by a vanishing first derivative of the energy function and represents the minimal energy value found in the considered range of the attachment angle. This feature of the second minimum does not influence, however, the conclusions of the analysis of the membrane fission conditions.

In the third regime, the affinity is in the range between the second and third characteristic values, 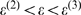. Under these conditions, the narrow neck is energetically more favorable (Fig. 4) and, hence, becomes stable whereas the broad neck turns quasi-stable.

Finally, in the fourth regime the affinity is larger than the third characteristic value, 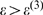. Here, the energy minimum corresponding to the broad neck vanishes and the only stable state of the system is that of the narrow neck.

The three characteristic affinity values, 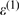, 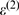 and 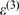, and the geometrical characteristics of the membrane bud in the four regimes of configurations are illustrated in the phase diagrams (Fig. 5a,b,c). The first two phase diagrams represents the total energies (Fig. 5a) and the corresponding attachment angels (Fig. 5b) of the broad and narrow neck configurations for a specific value of the membrane area 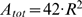. The third phase diagram (Fig. 5c) shows how 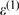, 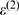 and 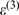 depend on the membrane area 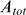 and, hence, on the area of a vesicle which would form if fission occurs. All the three characteristic affinities decrease with the membrane area 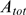 which means that the larger the membrane, the lower affinities are needed for generation of buds with narrow necks.

### Conditions for membrane fission

Recall that we analyze two requirements for membrane fission. According to the first requirement, the fission reaction has to be energetically favorable meaning that the total system energy in the post-fission state must be lower than in the fore-fission state, 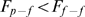. Upon this condition, the fission reaction may be slow because of the existence of kinetic barriers.

According to the second requirement, the energy barriers of the fission reaction must, practically, vanish, which guarantees fast rates of the membrane splitting. Particularly, the membrane neck has to narrow up to the threshold value 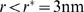, which guarantees that not just the overall fission reaction but also the intermediate hemi-fission stage is energetically favorable and does not limit the fission rate [35].

**Figure 6:**
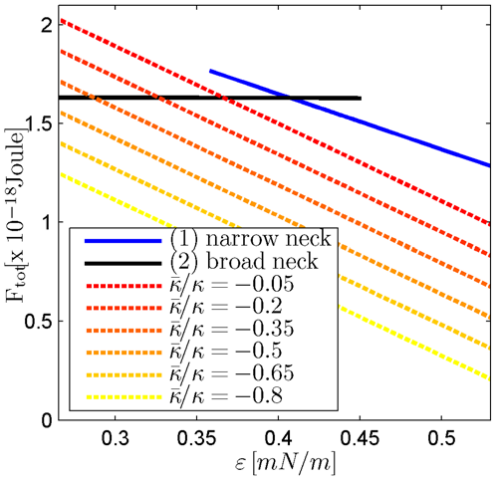
Comparison of the system energies in the fore- and post fission states for determination of the fission conditions. Dashed lines are the energies of the post-fission state for different values of the modulus of the Gaussian curvature; solid lines (1) and (2) represent, respectively, the energies of the narrow and broad neck configurations of the fore-fission state, 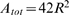.

**Figure 7:**
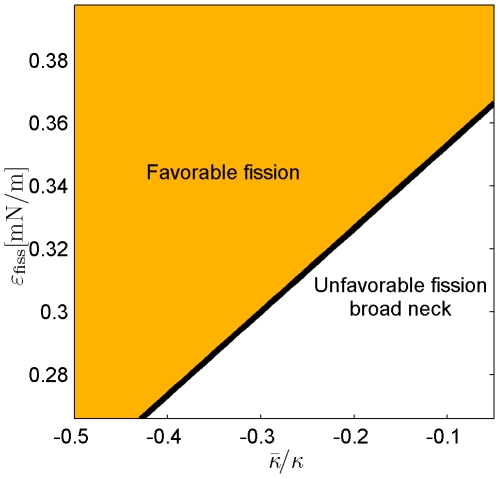
The affinity needed for fission of the broad neck configuration to be energetically favorable depending on the modulus of the Gaussian curvature. The total membrane area, 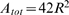.

The computed system energies in the fore- and post- fission states for different values of the affinity 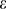 and different moduli of the Gaussian curvature 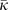 are presented in Fig. 6. According to these results the first requirement is always satisfied in the narrow neck configuration confirming the previous works. Also for the broad neck configurations the fission reaction may be energetically favorable. To this end the affinity 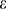 has to be larger than a certain value 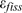 varying in the range between 0.27mN/m and 0.37mN/m for feasible values of the Gaussian curvature modulus 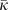 (Fig. 7). The more negative is 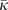, the looser are the fission conditions, i.e. the lower affinity 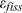 is needed for fission to be energetically favorable. However, to undergo fission from the broad neck configuration, the system has to overcome a substantial energy barrier and, in practical terms, the membrane splitting will not occur.

The requirement of fast fission can be fulfilled if the system reaches the narrow neck configuration. However, to achieve this state in the course of the membrane attachment to the protein dome, the system has to proceed through the whole range of the attachment angles beginning from 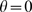 and up to 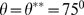. This means that the system has to move along one of the energy profiles represented in Fig. 4. According to Fig. 4, if the affinity value is smaller than 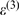, there is an energy barrier and the system has to overcome to reach the narrow neck configurations. This means that for 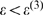 the membrane fission will be restricted kinetically. At the larger affinity values, 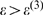, evolution of the membrane bud up to the narrow neck configuration is accompanied by a monotonous decrease of the energy and, hence, proceeds without kinetic restrictions. Summarizing, the condition for the fast fission is 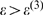.

### Experimental support for the model

**Figure 8:**
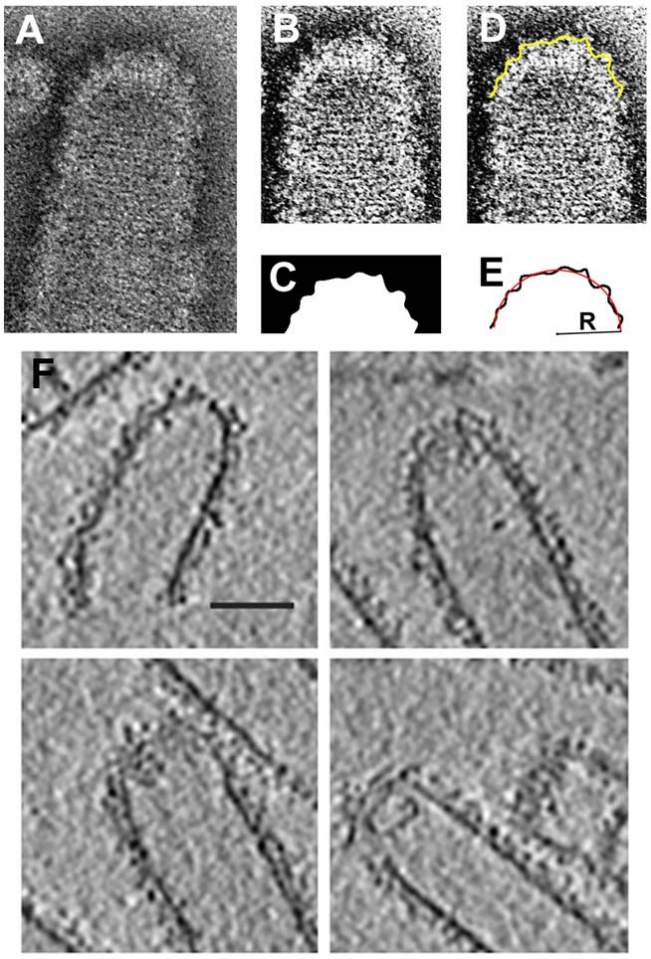
Imaging of the ESCRT-III (CHMP2A-CHMP3) assembly. (A) Electron micrograph showing an ESCRT-III tubule terminating into a hemispherical end-cap structure. (B) The closed end of the tubule after image processing. (C) Extracted edge of the ESCRT-III end-cap. (D) Fit of the extracted edge into the image. (E) Fit of a circle into the edge profile. The methods and experimental details for (A)–(E) are presented in [34]. (F) Cross sections of CHMP2A-CHMP3 end-capped tubular structures observed by cryo-electron tomography (see Materials and Methods). The images reveal the CHMP2A-CHMP3 protein layer and also the position of MBP proteins fused to the N-terminus of CHMP2A. The scale bar corresponds to a distance of 40nm.

To support the model, we studied the structures resulting from the CHMP2-CHMP3 self-assembly by negative staining [34] and cryo electron tomography (see Materials and Methods). We observed assembly of open tubes, tubes with flat closures, tubes with hemispherical almost closed ends (defects in closure) and closed tubular structures with hemi-spherical end-caps (Fig. 8). The presence of closure defects observed in the structures assembled *in vitro* might be due to fact that they have been assembled in the absence of membranes. In the current model we propose that these structures assemble directly on membranes. Formation of the closed hemi-spherically capped tubes substantiates the existence of the protein domes which play the central role in the model. These structures should represent the final stage of CHMP2-CHMP3 polymerization and our model suggests that they are physiologically relevant.

## Discussion

We suggested and analyzed a mechanism by which a minimal ESCRT-III complex composed of the mammalian ESCRT-III proteins CHMP2A and CHMP3 can drive fission of membrane necks. The mechanism is based on the experimental results which demonstrate that CHMP2A and CHMP3 heterodimers self-organize into tubular assemblies some of which reveal closed hemispherical dome-like end-caps. The external surfaces of these assemblies have a considerable affinity to lipid bilayers containing acidic lipids.

The essence of the model is that a CHMP2-CHMP3 tube with a dome-like end-cap self-assembles in the neck of an initial membrane bud generated by a circular filament of a CHMP4 (the latter suggested in [27],[29],[30]) (Fig. 1a,b). The CHMP2-CHMP3 self-assembly is accompanied by membrane attachment to the dome surface which results in narrowing of the membrane neck as illustrated in (Fig. 1b and Fig. 2a). Because of the hemi-spherical shape of the dome, progression of the dome assembly and the concomitant membrane binding to its surface leads to thinning of the neck and accumulation of the elastic stresses within its strongly curved membrane. If a certain degree of the neck thinning is achieved, fission of the neck membrane accompanied by the stress relaxation becomes energetically favorable. The proposed mechanism entails containment of the ESCRT-III proteins towards the cytosolic side after fission, which is consistent with the observation that the ESCRT-III proteins have not been detected within intra-luminal vesicles of the MVBs.

Since both CHMP2A and CHMP3 interact with Vps4 [34],[49],[50], it is important to understand a possible role this protein can play in action of CHMP2A-CHMP3 complexes on membranes. Although the results by Hanson and colleagues [31] indicated that Vps4 might play an active role during the ESCRT-III driven membrane remodeling process, in vitro budding experiments with GUVs suggested that vesicle formation and fission occurred in the absence of Vps4, albeit it seems to accelerate the process [29]. We suggest that Vps4 could still play an important role other than disassembly of ESCRTs from membranes [51]. The hemispherical shape of the protein end-cap can be maintained only if the bending rigidity of the end-cap wall greatly exceeds that of the lipid membrane. In case the end-cap bending rigidity is similar to or smaller than that of the membrane, the top segment of the end-cap, which is not covered by the membrane, will flatten. This would result in a decrease of the membrane attachment angle 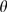 and, hence, hinder, to some extent, the membrane neck narrowing necessary for the neck fission. While this effect is small for the large degree of the membrane coverage corresponding to the narrow neck configuration, it can be considerable for the broad neck configuration, and may influence the probability of transition from the broad to the narrow neck. Given that the 4.5 nm thickness of the ESCRT-III shell [34] is, practically, equal to that of a lipid membrane (see e.g. [52]), the rigidity of the purely ESCRT-III complex might be not large enough to prevent flattening of the end-cap top. Strengthening of the ESCRT-III end-cap by binding of a Vps4 dodecamer, that exposes 12 CHMP binding sites on the inside of the ESCRT-III polymer, may provide the protein structure with an additional rigidity required for a more effective fission.

The neck fission results in formation of a separate vesicle and a hemi-spherical membrane cap covering the protein dome (Fig. 1c and Fig. 2b). Based on the model of membrane bending elasticity [42], we computed how large the membrane affinity to the protein dome has to be in order to enable fast fission of the membrane neck leading to formation of a separate vesicle. Below we discuss the feasibility of the obtained results for the affinity and show that the CHMP dome must be an efficient mediator of membrane fission.

### Membrane-protein affinity needed for fission

According to our computations, the affinity required to drive fission of the membrane neck depends considerably on the area of the membrane fragment undergoing budding and, hence, on the dimension of the vesicle generated in the result of fission (Fig. 5c). The ESCRT-III proteins have been implicated in generation of multivesicular bodies (MVBs) consisting of vesicles with characteristic diameters between 20 and 100 nm [19],[53] and in budding of enveloped viruses with diameters varying up to about 100 nm. Therefore, we performed calculations for the areas of the membrane bud 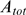 between 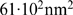 and 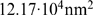 corresponding to the relevant range of the vesicle diameters.

The largest affinity denoted as 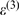 is needed to drive a kinetically unconstructed formation of a bud with a narrow neck of radius less 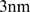 which enables fast fission. The affinity 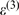 (as well as two other characteristic affinities, 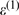 and 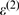, determining conditions for slower fission processes), decreases with increasing membrane area. The maximum value of 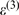 is needed for generation of the small 20 nm vesicles of MVBs. According to our results (Fig. 5c), the required affinity is 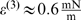.

The feasible values of the membrane affinity to the protein dome can be estimated based on a thermodynamic analysis of the kinetic measurements of the CHMP2A and CMHP3 monomer binding to the DOPS-SOPC bilayers [34]. According to these measurements, the CHMP2A and CHMP3 monomers dissociate from lipid with a dissociation rate constant (*k_off_*) of 0.08 s^−1^ and 0.3 s^−1^ respectively [34]. The association to lipid for both, CHMP2A and CHMP3, was found to be diffusion controlled thereby putting a lower limit on the association rate constant (*k_on_*) of 1×10^6^ M^−1^ s^−1^. The condition of equilibrium between the lipid-bound and free protein monomers resulting from the equality of the rates of their association to and dissociation from the lipid can be expressed by the equation
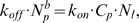
(4)where 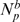 is the number of the lipid-bound protein monomers, 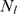 is the number of the lipid molecules and 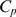 is the volume concentration of the free protein monomers. On the other hand, thermodynamically, the same equilibrium condition can be expressed through the equality of chemical potentials of the lipid-bound and free protein monomers,
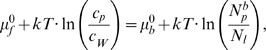
(5)where 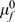 and 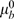 are the so called standard chemical potentials of the free and lipid-bound protein monomers accounting for the free energy of the direct monomer interaction with the surrounding, 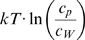 and 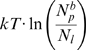 are the contributions of the free and lipid-bound protein monomers from the translational entropy in the solution and on the membrane surface, respectively, 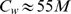 is the molar concentration of water molecules. Eq.5 takes into account that the whole lipid is organized into one or few extended membranes whose translational entropy has a vanishing effect on the chemical potentials.

The protein-membrane binding energy per protein monomer is related to the standard chemical potentials by 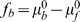, so that the affinity which represents, according to the definition above, an absolute value of the binding energy related to the unit area of the protein-membrane interface, is given by
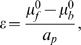
(6)where 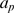
is the area of a CHMP monomer exposed to interaction with the membrane. Combining Eqs.4–6 we obtain for the affinity 
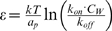
. Given the kinetic constants above, and the estimation for the monomer contact area 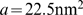 [33] we determine the membrane affinities of CHMP2A and CHMP3 to be 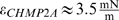 and 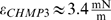. Taking into account that the protein dome consists of the CHMP2A-CHMP3 heterodimers, the average affinity should be about 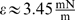, which exceeds almost by a factor of six the above estimation of 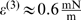 for the affinity required for fast fission of the 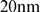 vesicles. Fission of larger vesicles requires even lesser affinities. Hence, the binding energy provided by the CHMP-membrane interaction must be excessively large and guarantees fast membrane budding and fission under all biologically relevant conditions.

### Conclusions

The suggested mechanism of membrane fission by the ESCRT-III proteins CHMP2A-CHMP3 and the related calculations demonstrate that dome-like assemblies of these proteins could scaffold membrane necks into strongly curved shapes and favor membrane fission. Since, in contrast to the proteins of the dynamin family, the ESCRT protein complexes attach the membrane to their external surfaces, the fission site emerges within a free membrane fragment aside of the zone of protein-lipid interaction. The task of the CHMP4 and CHMP6 subunits, which are recruited to the membrane upstream of the CHMP2 and CHMP3 recruitment, is to generate an initial membrane bud with a fixed membrane area whose neck has to undergo fission to complete the vesicle formation. A role for Vps4, in addition to its recycling function, can be in reinforcing the wall of the ESCRT-dome which facilitates membrane bending and fission. It is conceivable that the suggested mechanism is not limited by the action of ESCRT-III proteins but rather has a more general character.
